# Network complexity as a measure of information processing across resting-state networks: evidence from the Human Connectome Project

**DOI:** 10.3389/fnhum.2014.00409

**Published:** 2014-06-10

**Authors:** Ian M. McDonough, Kaoru Nashiro

**Affiliations:** Center for Vital Longevity, School of Behavioral and Brain Sciences, University of Texas at Dallas, Dallas, TX, USA

**Keywords:** functional connectivity, Human Connectome Project, information processing, multiscale entropy, neural complexity, resting-state networks

## Abstract

An emerging field of research focused on fluctuations in brain signals has provided evidence that the complexity of those signals, as measured by entropy, conveys important information about network dynamics (e.g., local and distributed processing). While much research has focused on how neural complexity differs in populations with different age groups or clinical disorders, substantially less research has focused on the basic understanding of neural complexity in populations with young and healthy brain states. The present study used resting-state fMRI data from the Human Connectome Project (Van Essen et al., 2013) to test the extent that neural complexity in the BOLD signal, as measured by multiscale entropy (1) would differ from random noise, (2) would differ between four major resting-state networks previously associated with higher-order cognition, and (3) would be associated with the strength and extent of functional connectivity—a complementary method of estimating information processing. We found that complexity in the BOLD signal exhibited different patterns of complexity from white, pink, and red noise and that neural complexity was differentially expressed between resting-state networks, including the default mode, cingulo-opercular, left and right frontoparietal networks. Lastly, neural complexity across all networks was negatively associated with functional connectivity at fine scales, but was positively associated with functional connectivity at coarse scales. The present study is the first to characterize neural complexity in BOLD signals at a high temporal resolution and across different networks and might help clarify the inconsistencies between neural complexity and functional connectivity, thus informing the mechanisms underlying neural complexity.

## Introduction

Research over the past decade has provided evidence that the temporal fluctuations of brain activity are crucial and fundamental properties of the human brain (e.g., Biswal et al., 2010; Deco et al., 2011; Wig et al., 2011). The synchrony, or correlation, of these temporal fluctuations between regions (i.e., functional connectivity) is one way to assess the communication of information in the brain and give rise to separate functional networks. These networks have been associated with specific cognitive processes (Seeley et al., 2007; Dosenbach et al., 2008; Vincent et al., 2008; Smith et al., 2009; Laird et al., 2011) and disruptions of these networks are associated with corresponding clinical disorders (e.g., Seeley et al., 2009; Posner et al., 2014). However, by concentrating efforts on understanding the correlations of temporal fluctuations, this research has largely ignored the *pattern* of these fluctuations within a region or network. Advances in signal processing methods have enabled the quantification of temporal patterns by assessing their randomness, or complexity (Costa et al., 2005). The more complex the pattern of brain activity, the more rich the information (e.g., Tononi et al., 1994, 1998; Garrett et al., 2013; Nakagawa et al., 2013) or more integrated the information (e.g., Vakorin et al., 2011; McIntosh et al., 2013) is within a network. Thus, whereas complex systems signify a normal and healthy state, highly regular (less complex) systems often mark dysfunction and disease (e.g., Pincus and Goldberger, 1994; Goldberger et al., 2002; Yang and Tsai, 2013). Consistent with this idea, measures of complexity have provided a useful tool for differentiating people of different age groups (e.g., McIntosh et al., 2008, 2013; Vakorin et al., 2011; Yang et al., 2012; Smith et al., 2013) and with different clinical disorders including Alzheimer's disease (e.g., Escudero et al., 2006; Mizuno et al., 2010; Yang et al., 2013b), autism (e.g., Bosl et al., 2011; Catarino et al., 2011; Ghanbari et al., 2013), attention deficit hyperactivity disorder (Gomez et al., 2013; Sokunbi et al., 2013), depression (e.g., Méndez et al., 2012), schizophrenia (Takahashi et al., 2010), tramatic brain injury (Beharelle et al., 2012), among others. As illustrated above, research is quickly demonstrating the utility of neural complexity as a marker of health, but our understanding is limited on how neural complexity is related to information processing and how it is characterized in a young, healthy system.

### Measuring neural complexity

Neural complexity is most often assessed using measures of entropy—the proportion of ordered patterns that can be detected in a signal (Pincus and Goldberger, 1994; Richman and Moorman, 2000; Lake et al., 2002). The fewer patterns that can be found within a signal (i.e., the more random the time series), the greater the signal complexity is. It has also been shown that recurring patterns within a physiological signal occur across a range of time scales (high to low frequencies) and so estimation of multiple time scales is necessary (Costa et al., 2005). Multiscale entropy (MSE) was developed to capture short-range/high frequency temporal complexity (i.e., fine scales) and long-range/low-frequency temporal complexity (i.e., coarse scales). Different time scales are calculated by down-sampling the original time series by averaging neighboring data points within non-overlapping windows. For a scale of 1, the original time series would remain intact. For a scale of 2, adjoining time points would be averaged, resulting in a time series half as long. It should be noted that the physiological nature of fine and coarse time scales remains unclear, but are thought to relate to different pathophysiological mechanisms (Mizuno et al., 2010; Yang et al., 2013b). One possibility is that fine time scales represent local information processing, while coarse time scales represent distributed information processing (e.g., Mizuno et al., 2010; Vakorin et al., 2011; McIntosh et al., 2013).

By using different time scales, MSE analysis can differentiate complex signals (assumed to carry meaningful information) from random noise (assumed to be unimportant). An increase in randomness of a signal does not always correspond to an increase in complexity because signals dominated by random fluctuations (e.g., white noise) are also highly irregular and should maximize entropy at fine time scales. However, completely random fluctuations should be characterized by decreases in complexity as time scales become coarser because these random fluctuations are smoothed out. In contrast, random fluctuations that carry 1/*f*^β^ spectral properties (e.g., pink or red noise) should exhibit a constant level of complexity across time scales due to their fractal properties (e.g., Costa et al., 2005; Smith et al., 2013). Hence, complexity represents an intermediate pattern of order and randomness that is only fully captured when measured across multiple time scales. Indeed, MSE analyses have shown a distinct pattern of complexity across time scales and this pattern differs from both white noise (rapid decline in complexity) and pink noise (constant level of complexity). Using EEG and MEG, neural complexity at fine time scales is characterized by relatively low complexity. As time scales become coarser, neural complexity increases and often plateaus or dips at the coarsest scales (i.e., a skewed inverted-U pattern). While fewer studies have investigated neural complexity of the BOLD signal, two recent fMRI studies have shown decreasing levels of complexity as time scale increases (using 5–10 total scales rather than 20–40 total scales; Yang et al., 2012; Smith et al., 2013). Thus, the pattern of neural complexity across time scales differs depending on the imaging modality used (EEG/MEG or fMRI), which in turn makes it difficult to compare findings across studies.

### Neural complexity: theories and evidence

While it is widely agreed that neural complexity is related to information processing, different theories have been proposed to explain how the two are related (for a recent review, see Garrett et al., 2013). A few of these theories are briefly summarized below.

#### A dynamic range of microstates

Neural complexity might represent the range or capacity of the brain to explore alternative brain states. Different resting-state models have proposed that fluctuations in brain activity operate in a balanced state of stable and synchronous patterns of activity (Honey et al., 2007, 2009; Ghosh et al., 2008; Deco et al., 2011; Shew et al., 2009, 2011; McIntosh et al., 2010). Nodes within a network transiently synchronize into sets of co-activated brain regions and noise within a system helps propel the brain into these different states. These ideas are consistent with proposals made by Friston et al. (2012), who argue that a characteristic feature of the brain is its tendency to wander, or not settle in to any particular state. By retaining an optimal degree of instability, a brain system can explore alternative hypotheses about the causes of incoming stimuli and permits the brain to learn by discovery (van Leeuwen, 2008). These wandering dynamics allow the system to converge on optimal responses to environmental demands. Admittedly, these ideas remain abstract, but the idea is that systems engaging in greater transition or exploration between different states (e.g., stable and synchronous states) have a greater propensity for information processing, thus increasing the level of complexity in a system.

#### Facilitation of neuronal firing

A more basic proposal is that the randomness of fluctuating brain activity represents a moderate level of noise in a system that enhances the probability of neuronal firing. Stochastic resonance models have illustrated that adding noise in a neural system can help subthreshold neurons reach firing thresholds (e.g., Wiesenfeld and Moss, 1995; Faisal et al., 2008; McDonnell and Ward, 2011). Moderate amounts of noise would lead to increased neuronal firing and, therefore, the potential for more information processing. Of course, too much noise would result in too much firing and a saturation of the signal. However, in this context there seems to be a balancing point where deviations from a moderate level of randomness (i.e., in a healthy system) could lead to abnormal firing rates, and potentially clinical disorders.

#### Regulation of neural synchrony

Rather than simply aiding neuronal firing, another proposal is that the degree of randomness in a system can facilitate or even inhibit the likelihood of synchrony between brain regions. Ghanbari et al. (2013) argued that a tightly regulated system is composed of brain activity low in complexity (i.e., predictable temporal patterns) possibly regulated by successful excitation and inhibition of glutamate and gamma-aminobutyric acid (GABA) neurotransmitters. More predictable signals (less neural complexity) establish an environment that facilitates phase relationships between brain regions, thus increasing the probability of synchrony, and in turn, information exchange across distributed brain regions. In contrast, more random signals (greater neural complexity) establish an environment in which phase relationships are difficult to obtain, thus decreasing the probability of synchrony and the amount of information exchanged across brain regions. Unlike previous proposals, this proposal suggests an inverse relationship with the degree of neural complexity and information processing. To the extent that different time scales are associated with different levels of complexity, the degree of synchrony across brain regions should differ between fine and coarse time scales.

Relatedly, neural models of information processing have suggested that both the degree of synchrony and time scale determine the maximum information transfer between neurons (Baptista and Kurths, 2008). Specifically, information processing should be maximized when neurons synchronize at coarse time scales (i.e., lower frequencies), but desynchronize at fine time scales (i.e., higher frequencies). Moreover, it has been suggested that coarse time scales reflect long-range interactions across distributed neural populations, while fine time scales reflect interconnectivity among local neural populations (Mizuno et al., 2010; Vakorin et al., 2011; McIntosh et al., 2013). Thus, the combination of these ideas would predict (1) less neural complexity is associated with greater synchrony between brain regions and (2) fine and coarse time scales should exhibit an inverse relationship (i.e., fine time scales should be characterized by low synchrony and greater information processing, and vice versa for coarse time scales).

#### Evidence from functional connectivity

The theories reviewed above all suggest that neural complexity is, in some way, related to neuronal dynamics that can either facilitate or inhibit patterns of neuronal firing, thus promoting information communication within local or across distributed neural populations. To the extent that these potential mechanisms facilitate information communication, neural complexity should be related to other established forms of information communication such as functional connectivity (see section Introduction). It could be argued that the first two theories (range of microstates and facilitation of neuronal firing) would predict a positive relationship between neural complexity and functional connectivity (i.e., greater neural complexity should be associated with greater functional connectivity). In regards to the last theory of neural synchrony, Ghanbari et al. (2013) make specific predictions of an inverse relationship between neural complexity and functional connectivity, while other researchers generally suggest that neural complexity should be positively associated with functional connectivity (e.g., Vakorin et al., 2011; McIntosh et al., 2013).

Evidence from EEG and MEG studies are able to capture a wide range of time scales to test (1) the extent that there is a relationship between neural complexity and connectivity, (2) the direction of this potential relationship, and (3) whether these effects differ at fine and coarse time scales. One of the first empirical demonstrations that neural complexity and functional connectivity are related was conducted by Mišić et al. (2011). Using graph theory metrics, they showed that the centrality of each node was positively associated with neural complexity at all-time scales. Centrality was measured by node degree (number of connections), regional efficiency (ease of accessibility from other nodes), and betweenness (the tendency to be in between other nodes). Interestingly, they found that this positive relationship was weaker at fine time scales, consistent with the idea that fine scales are dominated by more random signals, which decrease the likelihood of synchronization (Ghanbari et al., 2013). Consistent with these findings, McIntosh et al. (2013) investigated age differences in entropy and functional connectivity using both EEG and MEG. At fine scales, they found age-related increases in both complexity and within-hemisphere (i.e., local) connectivity. At coarse scales, the reverse occurred; they found age-related decreases in both complexity and across-hemisphere (distributed) connectivity.

However, Ghanbari et al. (2013) found different complexity-connectivity relationships when using MEG and children with autism spectrum disorders (ASD) compared with typically developing controls. In the frontal lobe/alpha-band signal, they found ASD-related decreases in complexity, but ASD-related increases in functional connectivity. In the temporal lobe/broad-band signal, they found ASD-related increases complexity, but ASD-related decreases in functional connectivity. These inverse relationships were similar across many different time scales and scalp electrodes in their study. Only one study fMRI study has investigated both neural complexity and functional connectivity within the same study (Yang et al., 2013a). This study investigated the effects of the gene Apolipoprotein E (APOE), a risk factor for Alzheimer's disease, on BOLD signals. They found that older adults with the APOE E4 gene had reduced neural complexity in the posterior cingulate and increased functional connectivity from this region to frontal regions. Because of the limitation of the sampling rate and length of fMRI time series, this effect can only be attributed to fine time scales. Note that in this study, the neural complexity and connectivity were never directly compared. Nevertheless, these latter two studies implicate an inverse relationship between neural complexity and functional connectivity.

### Limitations of prior studies

While complexity and connectivity are often correlated, the directions of these effects are not consistent. One issue is that the majority of studies investigating complexity-connectivity relationships have used populations at different ages or with clinical disorders rather than a population with a healthy and functional system. This method is problematic because the complexity-connectivity relationship could also be different in these populations. For instance, complexity-connectivity relationships could be positive in young adults, but negative in older adults. Additionally, these group differences might selectively impact fine or coarse time scales, further complicating interpretations.

Comparisons across studies also are made difficult by the different modalities used, which in turn affect the precision of the parameters used to calculate complexity, namely the sampling rate and length of the time series. For example, EEG and MEG have both shorter sampling rates and often have longer time series, thus enabling a more precise measure of both fine and coarse time scales. In contrast, the longer sampling rate and short time series in fMRI allow for only a few reliable time scales (at most) to be included in the analysis. The observed decrease in complexity across the time scales in fMRI suggests that complexity values at fine scales using fMRI might be similar to complexity values at mid or coarse scales using EEG or MEG. But of course, fMRI allows for a more precise localization of brain activity, thus being able to tie neural complexity to cognitive functions known to subserve specific brain regions or networks.

Lastly, few studies have attempted to test the degree that neural complexity differs between networks. Different resting-state networks (RSNs) are thought to represent large-scale component processes involved in different aspects of cognition (e.g., Seeley et al., 2007; Dosenbach et al., 2008; Vincent et al., 2008; Smith et al., 2009; Laird et al., 2011). Interestingly, because functional networks are often defined by the similarity of their time series, each brain region within a network should have the same pattern of neural complexity, but the level of complexity in each network might still differ from one another. These inter-network complexity differences might provide insight into the dynamics of each network including differences in local and distributed information processing.

### The present study

We addressed the limitations in prior studies in four ways. First, we aimed to capture a larger range of MSE in fMRI than has been used in previous studies by utilizing an fMRI time series with a very short sampling rate and long epochs within a scanning session. Specifically, data was used from the Human Connectome Project (HCP; Van Essen et al., 2013) in a set of 20 healthy young adults. The resting-state data from HCP consists of four resting state scans of 1200 time points each with a sampling rate of 720 ms. Not only will this sampling rate enable us to test whether patterns of MSE match those predicted by model simulations (e.g., Nakagawa et al., 2013), but this dataset also might allow a better comparison with studies investigating MSE in EEG/MEG, with the caveat that the slow hemodynamic response as measured via BOLD fMRI is fundamentally limited in temporal resolution. We predicted that a skewed inverted-U pattern would be found in neural complexity across time scales—the first fMRI study to show similar patterns of neural complexity patterns often found in EEG/MEG (e.g., Mišić et al., 2011; McIntosh et al., 2013; Yang et al., 2013b) and BOLD simulations (e.g., Nakagawa et al., 2013).

Second, we aimed to distinguish patterns of neural complexity from white, pink, and red noise. Few studies have attempted to estimate complexity from simulated noise and compare them to complexity values in BOLD signals. To the extent that each time scale has a complexity value that differs between the BOLD signal and simulated noise, those time scales are more likely to contain information related to neural signals, potentially subserving information processing. Additionally, simulating noise can address the null hypothesis that the pattern of complexity in BOLD signals differs from the pattern of complexity found in noise. While one study showed evidence that neural complexity in BOLD signals did not differ from white noise or from BOLD signals in white matter at fine time scales (Smith et al., 2013), we predicted that the higher sampling rate would allow for a better distinction between random noise (including white, pink, and red noise) and neural complexity in BOLD signals. In addition, we included BOLD signals from cerebral spinal fluid (CSF) as an additional test that neural complexity in gray matter differs from complexity in physiological signals presumably not neural in origin.

Third, using fMRI allows the characterization of neural complexity across different RSNs, which we refer to as *network complexity*. Network complexity was assessed in four key RSNs (default mode, cingulo-opercular, left and right frontoparietal), each of which has been implicated in higher-order cognition. For example, the default mode network (DMN) has been associated with a variety of cognitive domains including episodic memory, working memory, and self-reflection (e.g., Hampson et al., 2006; Buckner et al., 2008; Spreng et al., 2009). The cingulo-opercular network (CON) has been associated with the orientation/maintenance of attention and has been correlated with processing speed (e.g., Dosenbach et al., 2008; Menon and Uddin, 2010; Touroutoglou et al., 2012). The left and right frontoparietal networks (LFN and RFN) have been associated with the control of working memory, and decision making during goal-directed actions (e.g., Seeley et al., 2007; Dosenbach et al., 2008; Vincent et al., 2008). To the extent that information communication differs between RSNs, we predict that network complexity also would differ between RSNs at both fine and coarse time scales.

Lastly, the current study examined the relationship between neural complexity and functional connectivity in each of the RSNs to test our hypothesis that they would be related in all networks, but the complexity-connectivity relationship would depend on time scale. Critically, these analyses were conducted in a group of healthy young adults, thus providing a baseline from which to interpret other findings using populations of different ages or clinical disorders. Only one other fMRI study, to our knowledge, has assessed both complexity and connectivity in the same study, but no direct comparisons between the two measures were made, leaving open the question of how individual differences in the two measures might be expressed (Yang et al., 2013a).

## Materials and methods

### Participants

The first available 20 participants (22–35 years of age; 12 females) provided by the HCP (WU-Minn Consortium) were used in the present study. The HCP is a long-term study enabling the exploration of human brain circuits. These participants were unrelated to each other, relatively healthy individuals that were free of a prior history of significant psychiatric or neurological illnesses, but could have a history of smoking, heavy drinking, or recreational drug use without having experienced severe symptoms. All participants gave informed consent as approved by the Washington University in St. Louis institutional review board.

### fMRI acquisition

All data were acquired on a Siemens Skyra 3T scanner housed at Washington University in St. Louis. The scanner had a customized SC72 gradient insert and a customized body transmitter coil with 56 cm bore size (diffusion: Gmax = 100 mT/m, max slew rate = 91 mT/m/ms; readout/imaging: Gmax = 42 mT/m, max slew rate = 200 mT/m/ms). The HCP Skyra had the standard set of Siemen's shim coils (up to 2nd order) and used Siemen's standard 32 channel head coil. BOLD fMRI data were acquired using a T2^*^-weighted gradient-echo EPI sequence with 72 axial slices per volume, 104 × 90 matrix (2.0 × 2.0 × 2.0 mm^3^), FOV = 208 mm, TE = 33.1 ms, TR = 720 ms, FA = 52°. Across four scanning sessions, a total of 4800 frames were acquired.

Visual stimuli (i.e., a cross-hair) were presented and participant responses were collected using a Dell Optiplex 790 computer, running an Intel Core i3-2100 with 8GB of RAM and 64-bit Windows 7 Enterprise SP1. The E-Prime version was E-Prime 2.0 Professional Production Release (2.0.10.242). Visual stimuli were projected with a NEC V260X projector onto a lucite screen at 1024 × 768 resolution, and viewed by the participant using a mirror mounted on the top of the head coil. Participant responses were registered on a customized fiber-optic button box.

### Resting-state fMRI procedure

Scans from resting-state fMRI data were acquired in four scanning sessions of approximately 15 min each with eyes open relaxed and fixated on a projected bright cross-hair on a dark background presented in a darkened room. Across sessions, oblique axial acquisitions alternated between phase encoding in a right-to-left direction and phase encoding in a left-to-right direction.

### fMRI preprocessing

Postprocessed fMRI datasets were used in the present study, which consisted of standard processing methods using FSL (Jenkinson et al., 2002, 2012; http://fsl.fmrib.ox.ac.uk/fsl/fslwiki/). Below briefly summarizes the HCP processing pipeline (Glasser et al., 2013). First, gradient-non-linearity-induced distortion was corrected for all images. Next, FMRIB's Linear Image Registration Tool (FLIRT) was used for motion correction using the single-band reference (SBRef) image as the target. The FSL toolbox “topup” (Andersson et al., 2003) was used to estimate the distortion field in the functional images. The SBRef image was used for EPI distortion correction and is registered to the T1w image. One-step spline resampling from the original EPI frames to atlas space was applied to all transforms. Lastly, image intensity was normalized to mean of 10000 and bias field was removed. Additional processing steps were used to reduce variance unlikely to reflect neuronal activity using the REST toolbox (Song et al., 2011). These steps included removing linear trends and regression on six motion correction parameters and average BOLD signal using three masks included in the REST toolbox, which included a whole brain mask to remove global mean signal ^1^.

### Data analysis

#### Resting-state fMRI analysis

Dual regression analyses (Beckmann et al., 2009; Filippini et al., 2009) were implemented to isolate the time series within four major RSNs associated with higher-order cognition and to estimate subject-specific functional connectivity within each of the networks. Using this technique has several advantages over seed-based methods of identifying RSNs. Specifically, this method can help minimize the contribution of physiological-related signal to the resting-state network components (e.g., Kiviniemi et al., 2003; Beckmann and Smith, 2004), is much more robust to motion than seed-based methods (Calhoun and Adali, 2012), and a single time series can be extracted that is common across all voxels to be used to estimate network complexity.

In the dual regression analysis, we used four a priori templates from Smith et al. (2009) to reconstruct subject-specific time series and spatial maps for DMN (component 4), CON (component 8), LFN (component 10), and RFN (component 9). The first regression model used each template as a spatial predictor for the participant's 4 D data, producing a set of individual regression weights in the time domain (i.e., a time series for each spatial map). Using this time series as a temporal predictor for the 4 D BOLD data, the second regression equation estimated the individual regression weights in the spatial domain. These regression weights represented the degree that the time series in each voxel matched the time series for that component, and thus can be interpreted as a measure of functional-connectivity strength (e.g., Calhoun and Adali, 2012). Due to computational constraints, dual regression analyses were applied separately for each of the four scanning sessions instead of concatenating the four scanning sessions prior to computing the dual regressions.

The resulting subject-specific time series for each network of interest was used to calculate the network complexity of each RSN using multiscale entropy (see section Multiscale Entropy (MSE) Analysis) and averaged across the four scanning sessions. The resulting subject-specific spatial maps were Z-transformed and thresholded at *Z* > 3.29 (*p* ≤ 0.001). The strength of functional connectivity was estimated by averaging the thresholded *Z*-values within each network for each subject. The spatial extent of functional connectivity was estimated by counting the number of significant voxels within each network for each subject. Data was mapped onto the cortical surface using CARET software (Van Essen et al., 2001).

#### Multiscale entropy (MSE) analysis

After the time series from each RSN was extracted, the complexity of each network was estimated using MSE developed by Costa et al. (2005), which estimates sample entropy at different time scales. First, fine to more coarse-grained time series were created by down-sampling the original time series (i.e., averaging neighboring data points within non-overlapping windows). Second, sample entropy was estimated for the time series at each time scale (1–25 scales). Sample entropy is defined as the natural logarithm of the conditional probability that a given pattern of data of a specified length (*m*) repeats at the next time point for the entire time series at a given scale factor (of a dataset with a total length *N*). It considers subsequent patterns to be a repeat of the given pattern if they match within a certain tolerance (*r*) such that larger tolerance values increase the number of matches (Richman and Moorman, 2000; Lake et al., 2002). To the extent that a time series has a greater number of pattern matches, the time series is less random, and the entropy value is lower. In contrast, a smaller number of pattern matches is characterized as being more random, yielding a greater entropy value. We selected our parameters based on those used in prior studies investigating MSE using fMRI, *m* = 2 and *r* = 0.5 (e.g., Smith et al., 2013; Sokunbi et al., 2013). While exact parameters sometimes differ across studies, one of the advantages of using sample entropy (as used in MSE analysis) over other estimates of entropy (e.g., approximate entropy; Pincus, 1991) is that sample entropy is consistent over a broad range of possible *r*, *M*, and *N* values (e.g., Richman and Moorman, 2000; Lake et al., 2002; Sokunbi et al., 2013)^2^.

#### Simulations of neural complexity in noise and in CSF

Three profiles of noise were created and complexity in these noise profiles was compared with network complexity in each RSN. Patterns of complexity within brain signals should be different than those found in noise, potentially representing meaningful information processing. Time series consisting of white, pink, and red noise were estimated using f_alpha scripts (http://people.sc.fsu.edu/~jburkardt/m_src/colored_noise/colored_noise.html). White noise is a completely unpredictable signal with a constant power spectral density. Pink noise (1/*f* noise) and red noise (1/*f*^2^) are signals with a power spectral density that is inversely proportional to the frequency. Some evidence has suggested that the BOLD signal contains 1/*f* and 1/*f*^2^ spectral properties (e.g., Zarahn et al., 1997; Bullmore et al., 2004; Milstein et al., 2008; He et al., 2010; He, 2011) that are indicative of time points being temporally autocorrelated with more “noise” at some frequencies than others with this temporal autocorrelation potentially being time-lagged. Of course, the assumption is that some aspects of noise are not meaningful to information processing, even if the noise is structured. Three sets of simulated time series (white, pink, and red noise) were generated to match the length and variance of the time series of each participant for each network, totaling 640 time series (20 participants x four sessions x four networks × three types of noise). MSE analyses were then applied to each of the time series and averaged across the sessions and networks^3^.

BOLD signal from CSF also was extracted to evaluate the contribution of complexity within the BOLD signal that was non-neural in origin. We created a 3 mm sphere (23, −39, 14) in the posterior right ventricle to extract a time series (1) with minimal preprocessing (i.e., linear detrending only) and (2) with all of the preprocessing steps (e.g., detrending, regressing out movement, global signal, etc.).

#### Partial least squares (PLS) analyses

PLS was used in two sets of analyses to determine the extent that (1) neural complexity in each of the four RSNs differed from noise, (2) neural complexity was characterized by unique patterns in each of the four RSNs, and (3) neural complexity was associated with functional connectivity. PLS is a multivariate technique designed to identify latent factors that account for most of the variance in a data set (e.g., McIntosh et al., 1996). Because of the many time scales for each network, multivariate methods were an optimal way to capture how the pattern of network complexity in each RSN differed from noise, differed from each other, and was correlated with functional connectivity. For the first PLS analysis, the X matrix was organized in the form of (Subjects in Network × Time Scale), resulting in a 180 × 25 matrix that allowed for the comparison between neural complexity in the RSNs and simulated noise. The Y matrix corresponded to a dummy coding of the same (Subjects in Network × Time Scale) format.

For the second analysis, separate data matrices were created for each network. The X matrix was organized in the form of (Subjects × Time Scale), resulting in a 20 × 25 matrix that allowed for the comparison between networks/time scales with continuous values (i.e., behavioral PLS). The Y matrix was organized in the form of (Subjects × Functional Connectivity Values), resulting in a 20 × 2 matrix that consisted of functional connectivity values (strength and extent) for all subjects. These connectivity values were derived from the dual regression analysis. Because we were only interested in whether functional connectivity in a given network was related to neural complexity in that network (e.g., DMN functional connectivity to DMN neural complexity), four separate PLS analyses were conducted (one for each network).

For all PLS analyses, the cross-product of the X and Y matrices was then decomposed into a set of mutually orthogonal factors using singular value decomposition, resulting in a set of orthogonal latent variables (LVs). An LV consists of three components: (1) a singular value, (2) a vector of weights representing the pattern of time scales in the LV (i.e., salience values), and (3) a vector of weights representing the degree to which each subject expresses the given LV (i.e., brain scores). Brain scores were calculated by multiplying the salience scores by the neural complexity values for each subject.

Each LV was statistically evaluated two ways. First, we assessed the significance of the network contrast represented by a given LV by determining how different the contrast was from chance and from each other. To do this, we computed 1000 permutation tests in which conditions were randomly assigned within subjects. A measure of significance was calculated by estimating the proportion of times the permuted singular value was higher than the observed singular value. Second, to assess the reliability of the corresponding distribution across subjects (i.e., saliences), we resampled subjects within conditions (1000 bootstrap samples). A bootstrap ratio (BSR) was then calculated by dividing the saliences by the standard error of the generated bootstrap distribution. The bootstrap ratio is approximately equivalent to a z-score, whereby an absolute bootstrap ratio greater than 1.96 corresponds roughly to *p* < 0.05. For a given LV, positive bootstrap ratios supported the depicted contrast among conditions (e.g., A > B > C), whereas negative bootstrap ratios supported the inverse of the contrast among conditions (e.g., A < B < C).

## Results

### Resting-state networks

To verify that the dual regression analyses appropriately captured the four RSNs of interest, we averaged the subject-specific spatial maps (see Supplemental Figure 1). Visual inspection of these averaged maps indicated that the four networks of interest were successfully isolated.

### Neural complexity differs from noise

**Figure 1:**
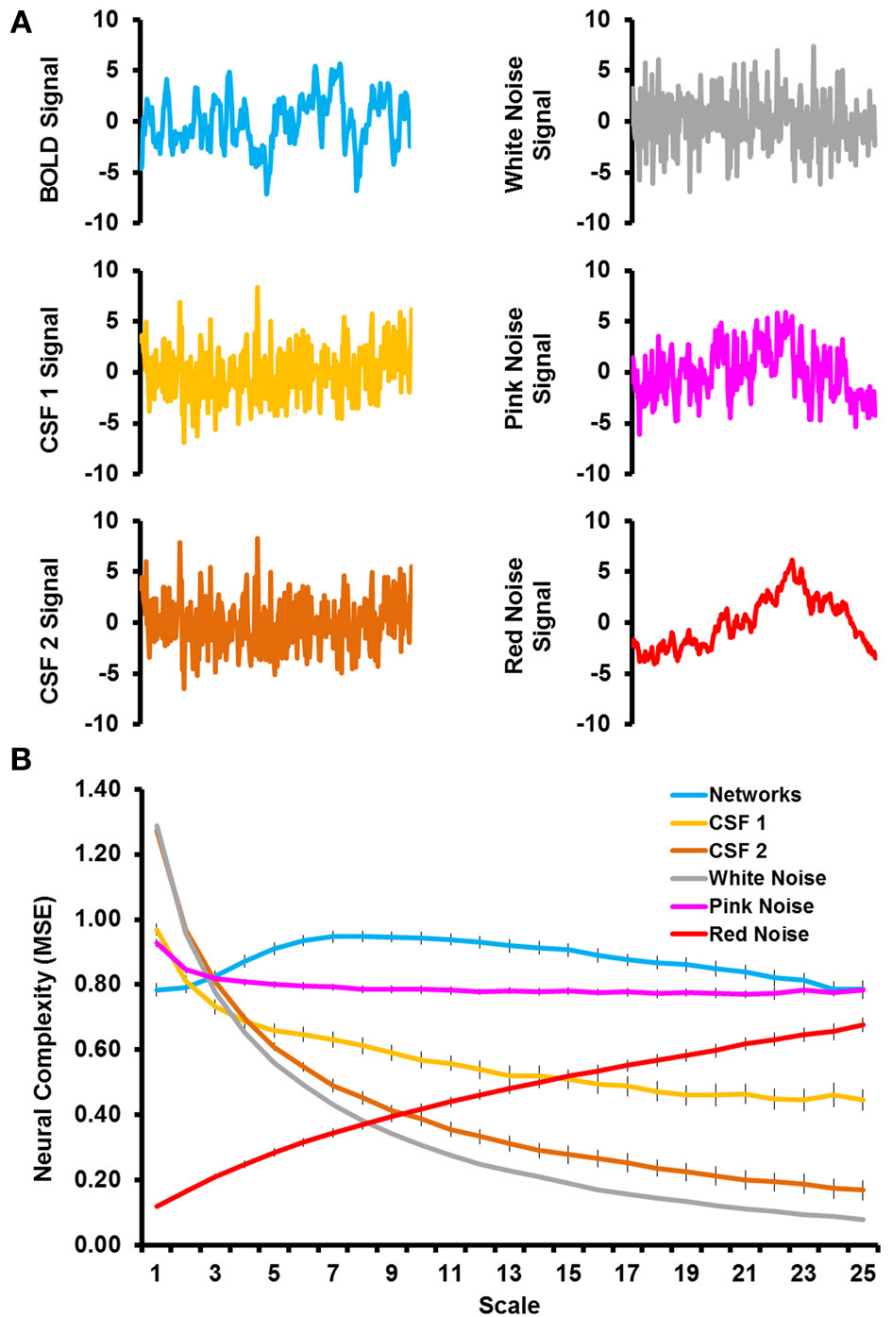
**(A)** Illustrates sample time courses from the first 300 time points for BOLD signal (top left), CSF signal that was detrended (middle left), CSF signal that was fully preprocessed (bottom left), simulated white noise (top right), simulated pink noise (middle right), and simulated red noise (bottom right). **(B)** Shows the results from the multiscale entropy analyses as a function of time scale for the grand mean of the resting-state networks (blue), CSF signals (light and dark orange), and simulated noise (gray, pink, and red).

Figure 1A shows sample time courses from the BOLD signal, CSF signal, and noise simulations and Figure 1B shows the complexity values from the MSE analyses. The RSNs were characterized by an increase in network complexity from fine to mid time scales, followed by a slight decline in network complexity as the time scale became coarser. Even when matched on time series variance and length, the complexity of simulated noise showed very different patterns from the RSNs. White noise was characterized by a very large degree of complexity at fine time scales, which decreased exponentially with coarser time scales. Pink noise was characterized by a relatively flat level of complexity across most of the time scales with the exception of a slight increase in complexity at the first time scale. Red noise was characterized by very low level of complexity at fine time scales and a steady increase as the time scales became coarser. The CSF signal that underwent all processing steps (labeled CSF 2) showed complexity values almost identical to the white noise simulations. The CSF signal that was only detrended (labeled as CSF 1) also showed a similar pattern as white noise, but the complexity values were slightly lower at fine time scales and higher at coarse time scales, resulting in a flatter slope. Thus, the patterns of network complexity qualitatively differed from those expected due to white, pink, and red noise. In addition, network complexity differed from other physiological signals of non-neural origin (i.e., in CSF) regardless of the type of preprocessing that was conducted.

**Figure 2:**
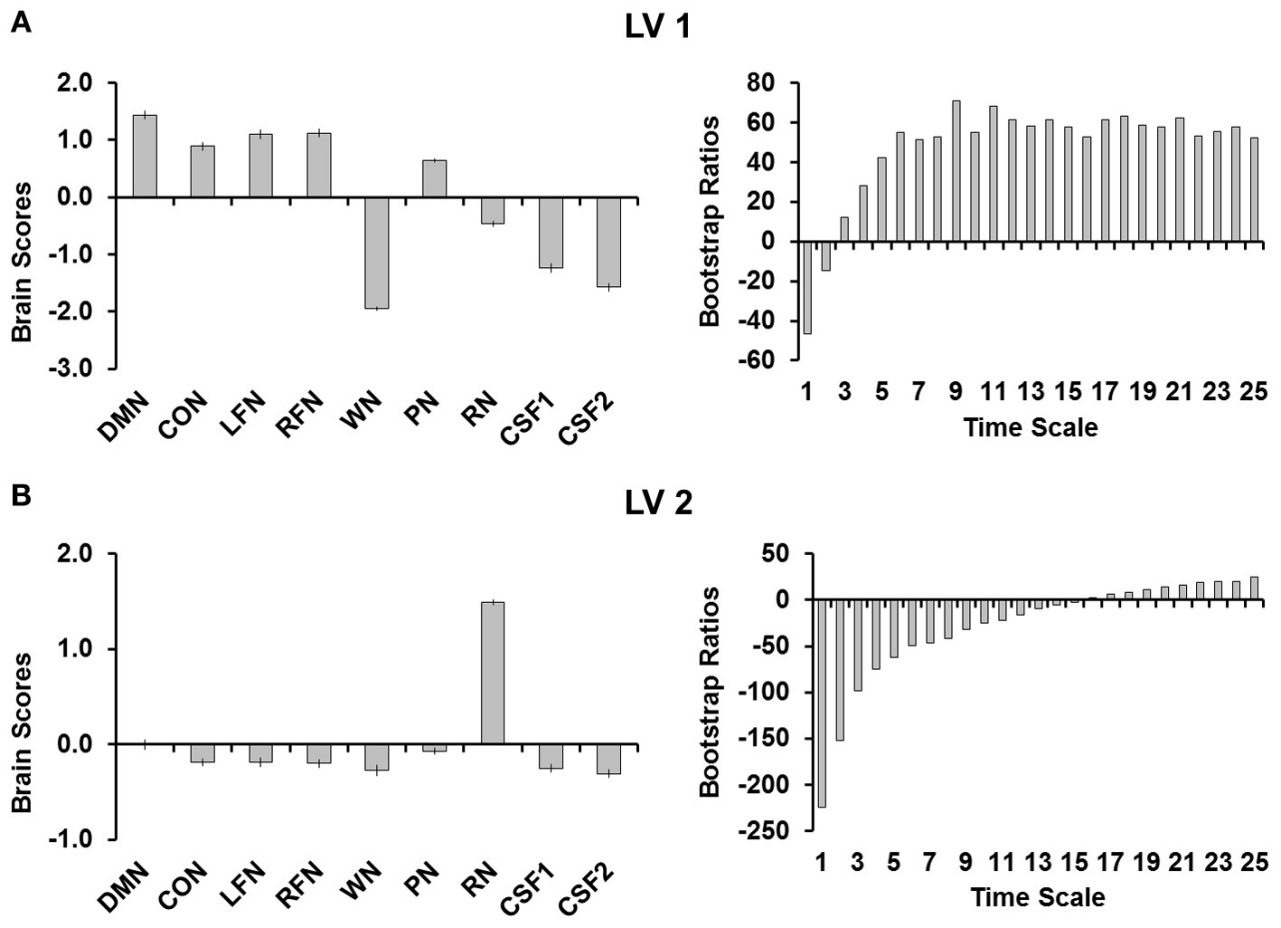
**(A)** Shows the first LV and **(B)** shows the second LV from the PLS analysis. Brain scores are plotted on the left with error bars representing confidence intervals and the bootstrap ratios are plotted on the right to evaluate the stability of the relationships for each of the time scales. LV 1, first latent variable; LV 2, second latent variable; DMN, default mode network; CON, cingulo-opercular network; LFN, left frontoparietal network; RFN, right frontoparietal network; WN, white noise; PN, pink noise; RN, red noise; CSF1, cerebro-spinal fluid with signal detrended; CSF2, cerebro-spinal fluid signal after all preprocessing steps were completed.

PLS analyses were conducted to quantify the differences between network complexity in the RSNs, complexity in the simulated noise, and complexity in CSF. This analysis revealed two significant LVs (both *p*'s < 0.001; Figure 2). The first LV (Figure 2A) explained 83.86% of the covariance and separated the RSNs from all other signals (i.e., noise and CSF) except for pink noise. Although pink noise represented a pattern more similar to the RSNs, the brain scores revealed that complexity in pink noise also significantly differed from each of the RSNs. The bootstrap ratios indicated that the RSNs differed from noise at each time scale, with the third scale showing the smallest (but still reliable) difference (BSR = 12.05). The second LV (Figure 2B) explained 15.71% of the covariance and separated red noise from the other signals. The bootstrap rations indicated that this difference occurred at each time scale except time scale 16 (BSR = 1.88).

### Network complexity differs between RSNs

**Figure 3:**
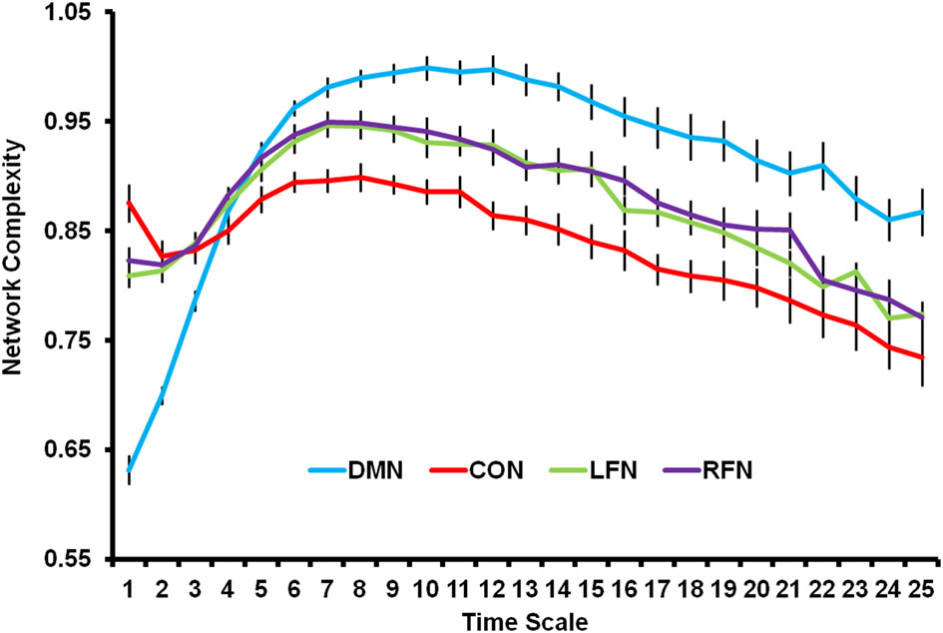
**Network complexity is plotted across time scales for the DMN, CON, LFN, and RFN**. All networks showed a skewed inverted-U pattern of neural complexity across the time scales. Neural complexity at fine time scales was greatest for the CON, followed by the LFN and RFN, and was smallest for the DMN. However, neural complexity at mid and coarse time scales was greatest for the DMN, followed by the LFN and RFN, and was smallest for the CON. DMN, default mode network; CON, cingulo-opercular network; LFN, left frontoparietal network; RFN, right frontoparietal network. Error bars represent standard error of the mean.

The previous PLS analysis also indicated that each of the four RSNs differed from each other. As shown in Figure 3, network complexity was smallest in the DMN at fine time scales, but network complexity was greatest in the DMN at mid and coarse time scales relative to the other networks. The CON showed the opposite pattern; network complexity was largest in this network at fine time scales, but smallest at mid and coarse time scales. The LFN and RFN did not differ from each other, and fell in between the DMN and CON. The present results suggest that RSNs show different temporal network dynamics. One possibility, for instance, is that the DMN is characterized by less local processing and more distributed processing relative to the other networks.^4^

### Network complexity is associated with functional connectivity

#### Default mode network

**Figure 4:**
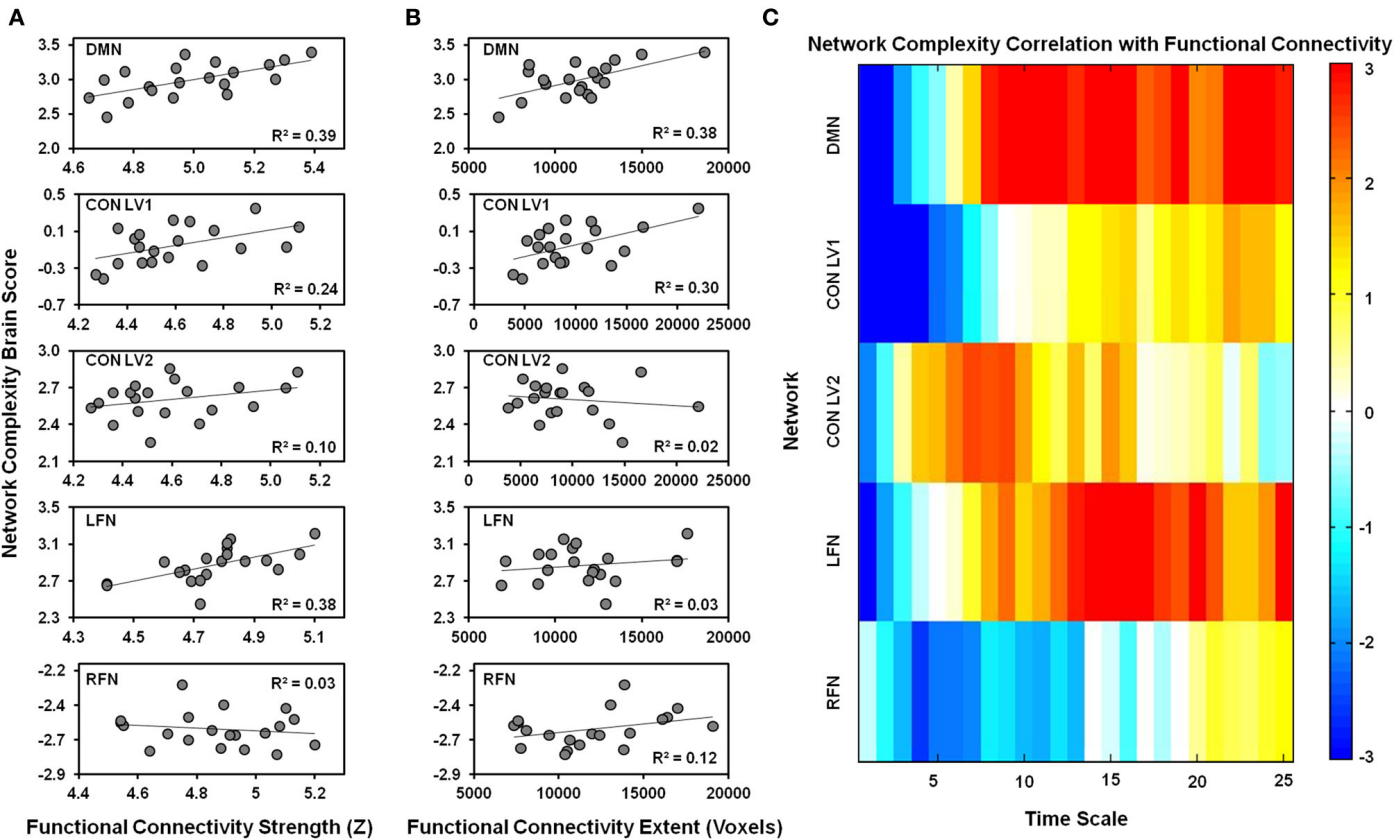
**The relationship is plotted between network complexity and functional connectivity. (A)** Shows the correlations between functional connectivity strength (x-axis) and the network complexity brain scores from the PLS analysis (y-axis). **(B)** Shows the correlations between functional connectivity extent (x-axis) and the network complexity brain scores from the PLS analysis (y-axis). The brain scores represent the pattern of network complexity across time scales as shown in **(C)**. Cool colors represent a negative association and hot colors represent a positive association between functional connectivity and network complexity at a given time scale. Network complexity within each network exhibits a similar relationship with functional connectivity; negative relationships were found at fine time scales and positive relationships were found at coarser time scales. DMN, default mode network; CON, cingulo-opercular network; LFN, left frontoparietal network; RFN, right frontoparietal network; LV1, latent variable 1; LV2, latent variable 2.

One LV was significant (*p* = 0.006), indicating that network complexity was associated with functional connectivity in the DMN (Figure 4). The Pearson-correlation values were significant for both functional-connectivity strength [*r* = 0.63, 95% CI (0.49, 0.82)] and functional-connectivity extent [*r* = 0.62, 95% CI (0.35, 0.83)]. The salience values from the LV (Figure 4C) indicated that these correlation patterns were characterized by a negative association between network complexity and functional-connectivity strength and extent at fine time scales, but a positive association at coarser time scales. The bootstrap ratios indicated that these associations were reliable at time scales 1, 2, and 8–25.

#### Cingulo-opercular network

The first LV was marginally significant (*p* = 0.07), but the Pearson-correlation values were significant for both functional-connectivity strength [*r* = 0.49, 95% CI (0.34, 0.74)] and functional-connectivity extent [*r* = 0.55, 95% CI (0.38, 0.80)]. The salience values from the LV indicated that these correlation patterns were characterized by a negative association between network complexity and functional connectivity-strength and extent at fine time scales, but a positive association at coarser time scales. The bootstrap ratios indicated that these associations were reliable at time scales 1–6.

The second LV was significant (*p* = 0.03). The Pearson-correlation value was significant for functional-connectivity strength [*r* = 0.31, 95% CI (0.04, 0.66)], but not for functional-connectivity extent (*r* = −0.15, 95% CI (−0.60, 0.32)]. The salience values from the LV indicated that these correlation patterns were characterized by a negative association between network complexity and functional-connectivity strength at fine time scales, but a positive association at coarser time scales. The bootstrap ratios indicated that these associations were reliable at time scales 1, 6–9, and 15.

#### Left frontoparietal network

Only one LV was marginally significant (*p* = 0.08) and the Pearson-correlation value was significant for functional-connectivity strength [*r* = 0.61, 95% CI [0.50, 0.82)], but not for functional-connectivity extent (*r* = 0.18, 95% CI [−0.16, 0.54)]. The salience values from the LV indicated that these correlation patterns were characterized by a negative association between network complexity and functional-connectivity strength at fine time scales, but a positive association at coarser time scales. The bootstrap ratios indicated that these associations were reliable at time scales 1, 9, 12–21, and 25.

#### Right frontoparietal network

Only one LV was significant (*p* = 0.05). The Pearson-correlation value was not significant for functional-connectivity strength (*r* = −0.16, 95% CI [−0.44, 0.51)], but was significant for functional-connectivity extent (*r* = 0.34, 95% CI [0.20, 0.54)]. The salience values from the LV indicated that these correlation patterns were characterized by a negative association between network complexity and functional-connectivity extent at fine time scales, but a positive association at coarser time scales. The bootstrap ratios indicated that these associations were reliable at time scales 4–7.

#### Correcting for multiple-comparisons

To test whether network complexity was associated with functional connectivity, we conducted four separate PLS analyses. When correcting for multiple comparisons using Bonferroni correction, significance would be restricted to *p*-values < 0.013 (0.05/4). This stricter threshold rendered only the correlation with network complexity and the DMN significant. Nevertheless, the pattern was very similar across all networks, supporting for the robustness of the findings.

## Discussion

The present study aimed to better understand the complexity of brain signals in a young, healthy population using fMRI. By using a dataset with a fast sampling rate and long scanning sessions, we were able to show that neural complexity within the BOLD signal is characterized by a skewed inverted-U pattern across time scales. Specifically, neural complexity was characterized by less complexity at fine scales (i.e., more regular patterns of brain activity), a sharp increase at mid scales, followed by a slow decline in complexity as the time scale became coarser. This study is the first to show this pattern in the BOLD signal, but is often found in EEG, MEG and model simulations (e.g., Nakagawa et al., 2013). While previous fMRI studies were limited in their scope, making it difficult situate MSE in the BOLD signal with MSE in electrical activity, the present study revealed some similarities when a high sampling rate and longer epochs were employed. Furthermore, the present study expanded upon previous research by showing that neural complexity in the BOLD signal differed from noise, differed from non-neural signals, differed between RSNs, and was correlated with functional connectivity. We elaborate on these key findings below.

### Differentiating neural complexity from noise

Neural complexity was estimated by measures of sample entropy, or the degree of randomness within a signal. While greater complexity has generally been associated with better cognition (e.g., McIntosh et al., 2008; Sokunbi et al., 2011; Yang et al., 2012), health (Yang and Tsai, 2013), and maturing brain systems (e.g., McIntosh et al., 2008; Vakorin et al., 2011), signals of pure noise also are highly random. Thus, signals containing meaningful information must be differentiated from signals varying randomly. Few studies have compared random patterns of fluctuations with complex patterns within the BOLD signal. However, one fMRI study conducted by Smith et al. (2013) compared complexity values of pink and white noise with the BOLD signal. They found that pink noise was characterized by similar levels of complexity across all time scales (i.e., resembling a flat line), and this pattern largely differed from the BOLD signal, which showed an exponential decrease in complexity values from fine to coarse time scales. The patterns of pink noise in the present study resembled the Smith et al. (2013) study, showing similar levels of complexity across time scales, which differed from BOLD signal.

In contrast to pink noise, Smith et al. (2013) found that complexity values in white noise did not differ from those in the BOLD signal at fine time scales, but rather began to differentiate as the time scales became coarser. Also striking in their study was the similar pattern of complexity between white noise and the BOLD signal; complexity values started high and exponentially decreased at similar rates. While results from the present study also showed patterns of complexity in white noise that exponentially decreased with increasing time scales, complexity in white noise differed from neural complexity in the BOLD signal at each time scale. One clear difference between the present study and the study conducted by Smith et al. (2013) is the greater temporal resolution and longer sessions in the present study, allowing for more precise estimations of complexity. Another key difference was that Smith et al. (2013) averaged complexity values across all gray matter voxels, whereas the present study only assessed gray matter voxels within networks associated with higher-order cognition. Averaging across different networks may have led to a mixing of different patterns of neural complexity, and thus a different pattern from the current study. Along these lines, we did find evidence that different RSNs were characterized by significantly different levels of complexity at multiple time scales. The complexity patterns of white noise also resembled the complexity patterns within CSF, supporting the idea that complexity within RSNs are neural in origin. Lastly, this study is the first to compare simulations of red noise, which contains spectral properties similar to the BOLD signal (but with a different exponent than pink noise). Unlike white and pink noise, red noise differed the most from complexity in the RSNs at fine time scales and became more similar at coarse time scales. Together, these studies suggest that some time scales may be dominated by random noise rather than meaningful signal. Because acquisition parameters often differ across studies, assessing noise is important to make meaningful interpretations of neural complexity.

### Characterizing network complexity across RSNs

Neural complexity should differ between RSNs to the extent that the complexity represents the temporal dynamics within that system, possibly relating to the interconnectivity among local neural populations and long-range interactions across distributed neural populations (Mizuno et al., 2010; Vakorin et al., 2011; McIntosh et al., 2013). The most striking finding across networks was that the DMN showed the smallest degrees of network complexity at fine scales, but the largest degrees of network complexity at mid and coarse scales relative to the other networks. Because fluctuations of brain activity while at rest largely represent the history of co-activations between regions (e.g., Wig et al., 2011), this observation would suggest that the DMN consists of greater degrees of information processing across distributed connections (e.g., medial frontal and medial parietal regions) relative to other networks. In contrast to the DMN, the other networks did not have as large of a range of complexity across time scales, suggesting relatively similar levels of information processing among both local and distributed connections. Interestingly, the CON showed the greatest degree of network complexity at fine scales, but the smallest degree of network complexity at coarse scales.

The correlations between network complexity and functional connectivity can help understand how to interpret the differences in complexity between RSNs at fine and coarse time scales. The network complexity across all of the RSNs showed a consistent pattern; network complexity was negatively correlated with both the strength and extent of functional connectivity at fine time scales, but was positively correlated at coarse time scales. This pattern is most consistent with the proposal that neural complexity is related to the regulation of neural synchrony (section Regulation of Neural Synchrony) and with the ideas that that information processing is maximized when neurons desynchronize at fine time scales, but synchronize at coarse time scales, consistent with neural models showing that time scale is critical to understand the neural dynamics involved in information transfer between neurons (Baptista and Kurths, 2008). Thus, one interpretation of the present results is that the DMN shows both the greatest degrees of desynchrony at fine scales and synchrony at coarse time scales relative to the other networks.

This interpretation is consistent with the idea that regions within the DMN serve as hub centers that act as critical gateways for information processing that integrate diverse sources of information within local and across distributed networks (e.g., Mesulam, 1998; Bassett and Bullmore, 2006; Buckner et al., 2009). For example, the DMN also is associated with many domains of cognition, and therefore information is constantly being exchanged within and across this network. Indeed, the default mode network has been related to episodic memory, imagining the future, self-reflection, mentalizing, divergent thinking, working memory, reading comprehension, and constructing moral judgments (Hampson et al., 2006; Buckner et al., 2008; Li et al., 2009; Spreng et al., 2009; van den Heuvel et al., 2009).

The fact that the other RSNs showed greater network complexity at fine scales, but less network complexity at coarse scales may be related to network dynamics at rest compared with online processing. For instance, activity in the DMN is often elevated at rest, but suppressed during tasks—especially those requiring cognitive control (e.g., Shulman et al., 1997; Mazoyer et al., 2001; Raichle et al., 2001). This pattern is consistent with the current study in that the DMN shows the greatest range in neural complexity at rest. Because the CON, LFN, and RFN have been implicated in online processing (i.e., are task positive networks), it is possible that the range of network complexity might change during a task. Thus, depending on the exact task and degree of cognitive control exerted, these networks might show the greatest range in neural complexity (i.e., smallest level of complexity at fine scales, but greatest level of complexity at coarse scales). No study to our knowledge has assessed neural complexity using MSE at rest and during a task—a possible avenue for future work.

### Alternative interpretations of neural complexity

While the results from the present findings largely support the idea that neural complexity is involved in the regulation of neural synchrony (or asynchrony), other interpretations cannot be entirely ruled out. These other interpretations, however, require additional explanations or evidence that is outside the scope of the present study. Specifically, an alternative idea is that neural complexity might represent the range or capacity of the brain to explore alternative brain states (for review, see Garrett et al., 2013; see section A Dynamic Range of Microstates). This idea proposes that randomness in brain activity arises from the constant fluctuation or transition of different brain states. To the extent that these transitions create complex patterns and are associated with information processing, then only low frequency signals at coarse time scales represent these types of transitioning neural dynamics. This theory does not explain the negative correlations between network complexity and functional connectivity at fine time scales. Another idea is that the randomness of fluctuating brain activity represents a moderate level of noise in a system that enhances the probability of neuronal firing (for review, see Garrett et al., 2013; see section Facilitation of Neuronal Firing). The current findings are consistent with this idea to the extent that what constitutes a moderate degree of noise differs between fine and coarse time scales. Less noise (and thus more regular brain activity) would play an optimal role to facilitate neuronal firing at fine scales, but more noise (and thus more random brain activity) would facilitate neuronal firing at coarse scales. However, it is not clear how these different levels of noise would interact with time scales in this manner.

While more work still needs to be done to fully understand how neural complexity fits in with the larger context of neural dynamics, a few key questions remain outstanding. First, do different degrees of neural complexity establish an environment that facilitates functional connectivity as proposed by Ghanbari et al. (2013)? This idea suggests that neural complexity does not represent information processing, *per se*, but rather influences the conditions under which optimal information processing can occur. Specifically, Ghanbari et al. (2013) proposed that the synchrony between any two nodes is more likely when signals are more predictable, thus having less complexity. While our findings only support this idea at fine time scales, the possibility remains that neural complexity does not directly correspond to the degree of information processing.

On the other hand, it has been suggested that neural complexity does relate more directly to the richness of information (e.g., Tononi et al., 1994, 1998; Nakagawa et al., 2013) or the amount of information integration (e.g., Vakorin et al., 2011; McIntosh et al., 2013) within a network. Evidence supporting this idea comes from estimates of neural complexity during different task conditions. For instance, neural complexity has been found to be greater during conditions when eyes are open compared with conditions when eyes are closed (Hogan et al., 2012), when learned information is highly familiar compared with less familiar (Heisz et al., 2012), when retrieving episodic information compared with semantic information (Heisz et al., 2013), and when faces are processed inverted compared with upright (Mišić et al., 2010). This idea would suggest that neural complexity is directly influenced by online processing and dynamically changes depending on concurrent cognitive processes.

### Implications for aging and clinical disorders

One of the goals of the present study was to assess neural complexity in a population that was young and healthy to better understand the pattern of findings in populations of different ages and clinical disorders. A common notion is that greater neural complexity is associated with healthier and more functional states. Thus, it is inferred that a decrease in neural complexity is associated with clinical disorders (cf. Yang and Tsai, 2013). The present results speak against such broad conclusions. At fine time scales—which would include most fMRI studies—low levels of neural complexity might be optimal for maximum information processing. Specifically, we found that decreases in neural complexity at fine time scales were associated with greater functional connectivity in a healthy sample across four key networks associated with higher-order cognitive processing. Thus, increases in neural complexity at fine time scales might actually be associated with a deficit in information processing. This pattern can inform recent studies investigating the effects of neural complexity and functional connectivity in populations at risk for developing Alzheimer's disease. For instance, a recent fMRI study showed that older adults who carried the APOE E4 allele—a risk for developing Alzheimer's disease—had less neural complexity in precuneus and posterior cingulate than non-carriers and also had increased functional connectivity in these regions with frontal regions compared with non-carriers (Yang et al., 2013a). Because young, healthy adults also show this relationship, it might be inferred that the decreases in neural complexity is associated with more information processing, potentially as a compensatory mechanism to counteract structural or other neurobiological declines (cf. Park and Reuter-Lorenz, 2009) stemming from having the APOE E4 allele (e.g., Poirier et al., 1993; Bookheimer et al., 2000; Small et al., 2000; Mahley et al., 2006). This interpretation differs from the general notion that decreases in neural complexity is associated with dysfunction in a system.

Other studies using EEG and patients with probable Alzheimer's disease also have shown decreases in neural complexity at fine scales. Furthermore, increases in neural complexity at coarse scales relative to healthy controls are evident (e.g., Escudero et al., 2006; Mizuno et al., 2010; Yang et al., 2013b). In light of the present findings, both the decreased levels of neural complexity at fine scales and the increased levels of neural complexity at coarse scales would be interpreted as corresponding to increased levels of functional connectivity. However, one assumption to this line of reasoning is that greater connectivity is always beneficial. While often times it might be beneficial—especially if healthy, young adults show a similar pattern—increases levels of connectivity might be detrimental (i.e., hyper connectivity; e.g., Bai et al., 2011; Mayer et al., 2011; Fornito et al., 2012). By relating both neural complexity and functional connectivity measures to behavioral performance, one can better assess whether these differences are associated with compensation or dysfunction.

A related and more neutral interpretation of neural complexity differences (without assuming increases or decreases are necessarily associated with a dysfunctional system) is that greater neural complexity at fine scales is associated with a bias away from interconnectivity within local connections, whereas greater neural complexity at coarse scales is associated with a bias toward distributed connectivity between long-range connections. Similar arguments have been made in the context healthy aging. For example, McIntosh et al. (2013) investigated age differences in MSE and functional connectivity using both EEG and MEG. At fine scales, they found age-related increases in both complexity and within-hemisphere (i.e., local) connectivity. At coarse scales, the reverse occurred; they found age-related decreases in both complexity and across-hemisphere (distributed) connectivity.

### Limitations

There are some limitations in directly comparing these findings to studies using MSE in different imaging modalities. While the pattern of complexity in the current study is similar to that found in EEG and MEG studies, the origin of the brain signals still differs between modalities (i.e., electrical vs. BOLD activity) and thus the neural dynamics underlying complexity across time scales might also differ. For example, the study comparing young and old adults using EEG and MEG conducted by McIntosh et al. (2013) found both age-related increases in neural complexity and functional connectivity at fine time scales, which suggests a positive relationship between these two measures. If a positive relationship does exist between these measures in EEG and MEG, then neural complexity may be tapping into different underlying mechanisms than neural complexity in fMRI. While this might indeed be the case, that study did not directly correlate the two measures together, thus it could be the case that while aging does affect the overall level of neural complexity and connectivity, individual differences within each age group could still show a negative relationship.

Another limitation is that very few studies have analyzed MSE in fMRI, making the underlying neural mechanisms of MSE unclear. As outlined in the Introduction (section Neural Complexity: Theories and Evidence), differences in network complexity could be related to the range of microstates, facilitative noise in a system, or neural synchrony (local and distributed information processing). While we favor an interpretation of neural synchrony (for discussion, see section Alternative Interpretations of Neural Complexity), it is still possible that the type of “local processing” captured at fine time scales in EEG/MEG might be qualitatively different from the type of “local processing” captured at fine time scales in the current study. Nevertheless, the current study was able to capture a large range of time scales comparable to most EEG/MEG studies, suggesting that the relative differences in local vs. distributed processing remain similar. This idea is supported by the pattern of MSE that shows a low-value of network complexity at fine time scales, followed by a rapid increase in network complexity—a similar pattern to that found in EEG and MEG studies, suggesting that we might be tapping into local processing. Future research is needed to verify that the underlying network dynamics captured by MSE is similar across different imaging modalities.

## Conclusion

Neural complexity provides novel insights into the neural dynamics underlying information processing, but the neural complexity across different RSNs in healthy young adults has largely been ignored. The present study provided evidence that the complexity within BOLD signals differs from random fluctuations associated with noise, differs between RSNs, and that complexity is associated with both the strength and extent of functional connectivity across RSNs. These findings complement other analysis techniques aimed to measure information processing and can help better understand both healthy brain systems and abnormal brain systems including aged and clinical populations. Contrary to growing beliefs, less neural complexity is not always indicative of declines in information processing. Only by further exploring the brain's temporal dynamics across a variety of contexts will we come to fully understand neural complexity and its relationship with cognition and pathological states.

### Conflict of interest statement

The authors declare that the research was conducted in the absence of any commercial or financial relationships that could be construed as a potential conflict of interest.

## Footnotes

^1^Global signal regression is controversial because doing so can sometimes remove signal of interest, increase spurious long-range connections, and create “anti-correlated” networks (e.g., Murphy et al., 2009; Saad et al., 2012). However, removing global signal regression has also been shown to remove artifactual correlations due to movement (e.g., Satterthwaite et al., 2013). While more research needs to be conducted to verify instances when global signal removal is appropriate, the goals of the present study were to capture a time series representative of large-scale brain networks, while reducing the contribution of movement-related noise to these estimates. However, systematic investigations regarding the impact of removing global signal on MSE estimates are necessary to fully understand its impact on these MSE estimates.

^2^We should note that two studies (Yang et al., 2012, 2013a); used *m* = 1 rather than *m* = 2. We chose not to use *m* = 1 for several reasons. First, most studies employing MSE (across imaging modalities) use *m* = 2, thus allowing for more comparable parameters across studies. Second, instead of choosing parameters that minimize estimation error as recommended by Lake et al. (2002). Yang et al. (2012), (2013a); determined their parameters based on the number of regions that differed between higher and lower performing older adults. This criterion is potentially problematic because it was determined in a group of participants already showing cognitive decline, was not shown to statistically differ from other values (e.g., *m* = 2), and being able to show greater differences between groups does not guarantee that those parameters are less free from error or bias. Lastly, on a more conceptual level, finding a pattern of two consecutive points (when using *m* = 1) across a time series would qualify as a weak test of repeating “patterns” whereas finding a pattern of three consecutive points (when using *m* =2) would qualify as a stronger test of repeating patterns.

^3^Inspection of the noise simulations showed almost identical MSE patterns across the different networks. To test whether the noise simulations differed as a function of network, we conducted a 3 (Network) × 25 (Time Scale) repeated-measures analysis of variance separately for each noise type (white, pink, and red noise). In each of these analyses (not correcting for multiple comparisons), we found no main effects of network (all *p*'s > 0.18) and no network × scale interaction (all *p*'s > 0.74). Thus, the MSE results were averaged across the simulated networks within each subject, resulting in 25 white noise, 25 pink noise, and 25 red noise entropy values for each subject (each value corresponds to a single time scale).

^4^One might argue that differences in MSE across networks could be driven by differences in variance between the networks. Indeed, when calculating the variance using standard deviation (BOLD SD) in a repeated-measures ANOVA, we found a significant effect of network, [*F*_(3, 57)_ = 61.94, MSE = 0.138, *p* < 0.001], such that BOLD SD in DMN > LFN > CON = RFN. Interestingly, while the general patterns were similar to the MSE results (the range of MSE across time scale was largest for DMN and smallest for CON), LFN and RFN did not significantly differ in MSE, but did differ in BOLD SD, [*t*_(19)_ = 4.80, *p* < 0.001]. This deviation between MSE and BOLD SD in conjunction with the findings that the simulated noise data did not differ across networks (despite having differences in variance), suggests that variance is not driving the differences in MSE between networks.

## Supplementary material

Figure 1 **The four resting-state networks of interest were successfully isolated using a priori templates in dual regression analyses**. DMN, default mode network; CON, cingulo-opercular network; LFN, left frontoparietal network; RFN, right frontoparietal network.
