# Supplementary figures and images for: Network complexity as a measure of information processing across resting-state networks: evidence from the Human Connectome Project
Source: Front Hum Neurosci. 2014 Jun 10;8:409. doi: 10.3389/fnhum.2014.00409 (PMC4051265; doi:10.3389/fnhum.2014.00409)

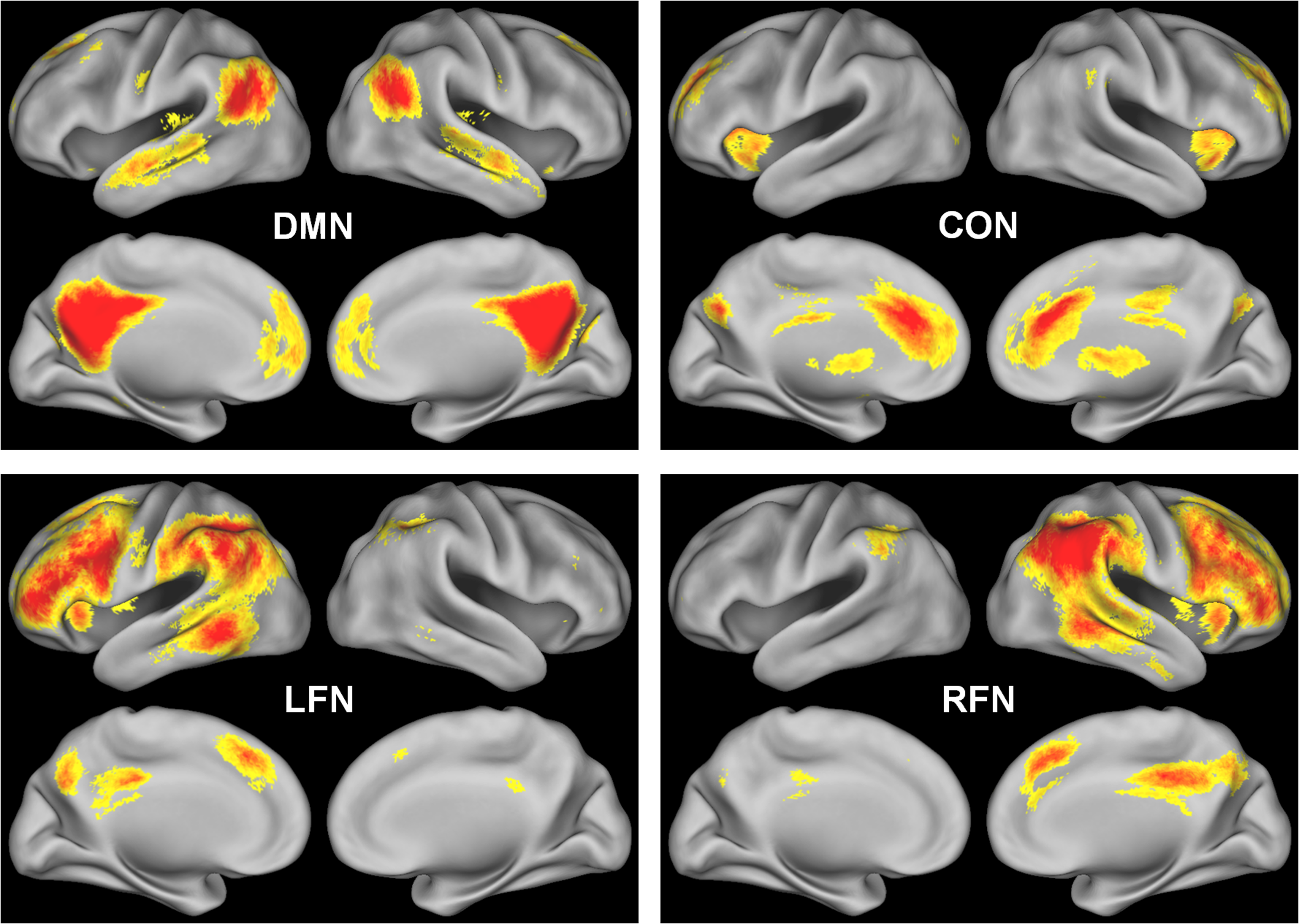

Supplement: Figure 1 — The four resting-state networks of interest were successfully isolated using a priori templates in dual regression analyses. DMN, default mode network; CON, cingulo-opercular network; LFN, left frontoparietal network; RFN, right frontoparietal network. [file Presentation1.ZIP › Supp Fig 1.TIF]
